# Dentists' decisions regarding the need for cuspal coverage for endodontically treated and vital posterior teeth

**DOI:** 10.1002/cre2.185

**Published:** 2019-04-15

**Authors:** Motasum Abu‐Awwad

**Affiliations:** ^1^ Department of Prosthodontics, School of Dentistry University of Jordan Amman Jordan

**Keywords:** cuspal coverage, decision making, endodontically treated teeth, posterior teeth, vital teeth

## Abstract

**Objectives:**

Deciding when cuspal coverage is needed for posterior teeth is considered a challenge for dentists. The aims were to assess dentists' decision making regarding the need for cuspal coverage for vital teeth (VT) and endodontically treated teeth (ETT) with varying amounts of tooth structure loss and to identify clinical situations of dissimilarity and uncertainty in decision making.

**Materials and Methods:**

A random sample of 182 dentists were invited to participate in the survey. The survey included photos of 13 posterior teeth: six VT and seven ETT. The clinical situations selected were based on a hypothetical scale of typodont teeth with ascending amounts of tooth structure loss. A brief description of each situation was provided. Each dentist was asked to decide whether cuspal coverage is needed, not needed, or unsure. Descriptive analyses using SPSS were conducted. Seventy‐five percent was chosen as a cutoff point for assessing similarity in decision making. The unsure answer reflected uncertainty. Associations were assessed using chi‐square test.

**Results:**

One hundred twenty dentists participated (65.9% response rate, 70 females). Median for years of experience was 3.5 (interquartile range 1.1–10.8). Analyses revealed a similarity percentage of <75% in decision making among dentists for six clinical situations: four VT and two ETT. More similarity was observed for situations at both ends of the scale with minimal and severe amounts of tooth structure loss and more for ETT than for VT. The highest percentages of uncertainty were more for VT than for ETT. Clinical conditions of VT were more likely to receive the “not sure” decision compared with those of ETT (*χ* ^2^, *P* < .001). No association was detected with gender (*χ* ^2^, *P* = .509) or years of experience (*χ* ^2^, *P* = .223).

**Conclusions:**

Dissimilarity and uncertainty in deciding when cuspal coverage is needed were observed especially for VT and teeth with a moderate amount of structure loss.

## 1 INTRODUCTION

Decision making regarding the best management for endodontically treated teeth (ETT) and vital teeth (VT) can be challenging for dentists (Cheung, 2005; Morgano, Hashem, Fotoohi, & Rose, 1994; Robbins, 1990; Zarow, Devoto, & Saracinelli, 2009). Determining whether cuspal coverage is needed or not is a common clinical decision faced by dentists in their daily clinical practice (Afrashtehfar, Ahmadi, Emami, Abi‐Nader, & Tamimi, 2017; Afrashtehfar, Emami, et al., 2017; Afrashtehfar & Tamimi, 2017).

Full cuspal coverage refers to covering all of the posterior tooth cusps with direct or indirect restorative material. Deciding whether a tooth would best be restored with cuspal coverage or an intracoronal restoration will depend on many factors such as whether the tooth is endodontically treated or not (Afrashtehfar & Tamimi, 2017; Aquilino & Caplan, 2002), the amount and distribution of tooth structure remaining (Afrashtehfar, Ahmadi, et al., 2017; Afrashtehfar, Emami, et al., 2017; Nagasiri & Chitmongkolsuk, 2005), the type and amount of load applied on the tooth during function (Loney, Moulding, & Ritsco, 1995; Torbjörner & Fransson, 2004), the parafunctional habits of the patient (Nishigawa, Bando, & Nakano, 2001), the esthetic value of the tooth, and the knowledge and experience of the dentist (Burke & Lucarotti, 2009; Lucarotti, Holder, & Burke, 2005a).

Many studies in the literature have focused on the risk of fracture and the management of ETT. ETT do not differ from VT in their biomechanical properties (Sedgley & Messer, 1992) or their levels of hydration (Huang, Schilder, & Nathanson, 1992). The loss of tooth structure from caries and existing restorations could lead to a weakened state in both the ETT and the VT (Reeh, Messer, & Douglas, 1989). However, what makes the ETT more prone to fracture than the VT is the loss of structural integrity associated with the access cavity, which leads to an increase in the cavity depth and cuspal flexure (Hansen & Asmussen, 1990; Hansen, Asmussen, & Christiansen, 1990; Pantvisai & Messer, 1995). The loss of a protective feedback mechanism in the ETT could also be a contributor to the increased loads on ETT, which could lead to an increase in the risk of fracture. However, it could be argued that the protective feedback mechanism comes from the proprioceptive receptors in the periodontal ligament (Randow & Glantz, 1986).

Even though many of the evidence in the literature advocates full cuspal coverage for posterior ETT for better longevity and survival rates (Aquilino & Caplan, 2002; Assif & Gorfil, 1994; Ferrari et al., 2012; Goodacre & Spolnik, 1994; Robbins, 1990; Sorensen & Martinoff, 1984), there are other studies in the literature that reported good survival rates without cuspal coverage in situations where significant amounts of tooth structure remained (Hansen et al., 1990; Mannocci, Bertelli, Sherriff, Watson, & Ford, 2002; Nagasiri & Chitmongkolsuk, 2005; Scotti et al., 2015). Two recent systematic reviews on posterior ETT and VT have found the failure rate to be affected by the amount of remaining tooth structure and the type of treatment provided for each clinical situation (Afrashtehfar, Ahmadi, et al., 2017; Afrashtehfar, Emami, et al., 2017).

The ability of the dentist to identify the clinical situations in which cuspal coverage can be avoided has many advantages, such as reducing the time and cost of the treatment, preserving more amounts of tooth structure, and avoiding over treatment (Fedorowicz et al., 2012; Grembowski, Fiset, Milgrom, Forrester, & Spadafora, 1997; Rasines Alcaraz et al., 2014). Therefore, knowledge in this area would be of importance to the dentists, the students, and the educators (Shugars, Hayden, Crall, & Scurria, 1997).

The aims of this study were to assess dentists' decision making regarding the need for cuspal coverage for different posterior ETT and VT based on the amount of tooth structure loss and to identify clinical situations of dissimilarity and uncertainty in decision making.

## 2 MATERIALS AND METHODS

A randomly selected sample of 182 dentists were invited to participate in this cross‐sectional study. Each dentist received an invitation letter, an information sheet, and a consent form that explained the aims of the study and assured the dentists that the data collected will be completely anonymous.

The demographic data collected in the survey were gender and years of experience. The survey included 13 different clinical situations, all of posterior teeth, with different amounts of tooth structure loss. The photographs of the clinical situations were presented in a random sequence to the dentists. A small description regarding the vitality of the tooth was provided for each clinical situation. The dentists were asked to assume that all the clinical situations were in need of restorative work, free of cracks, and that none of these clinical situations were subjected to lateral occlusal forces or increased forces due to parafunctional habits, such as teeth grinding or bruxism.

Each dentist was asked to decide whether cuspal coverage is needed or not for each clinical situation. Cuspal coverage was defined as covering all of the posterior tooth cusps with direct or indirect restorative material. The dentists were asked to view the clinical situations and make the decisions in the manner they do in their daily clinical practice of dentistry. The options for each clinical situation were as follows: (a) cuspal coverage is needed, (b) cuspal coverage is not needed, or (c) unsure if cuspal coverage is needed or not.

**Figure 1:**
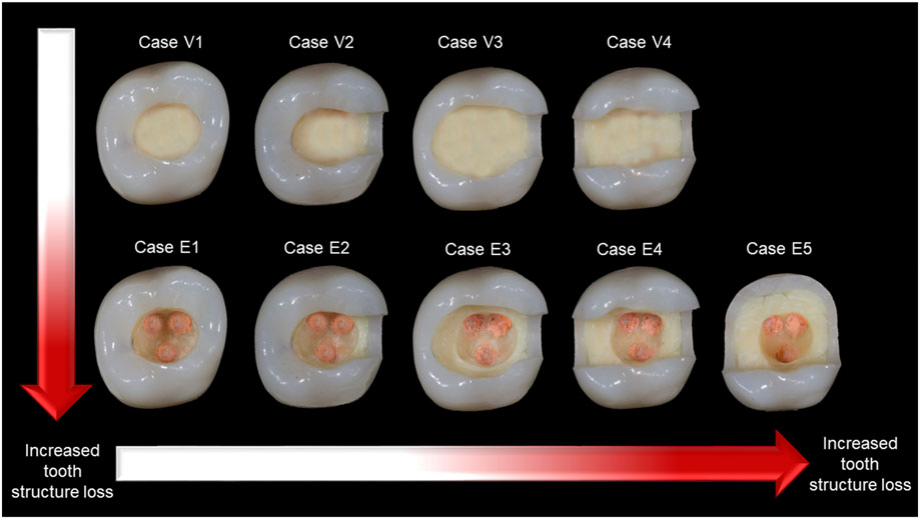
A hypothetical scale of different amounts of tooth structure loss prepared in the laboratory on typodont resin teeth. V refers to a vital tooth, and E refers to an endodontically treated tooth

The clinical situations that were included in the survey were selected based on a hypothetical scale prepared in the laboratory on typodont resin teeth. A systematic approach was used to prepare different cavities reflecting different amounts of tooth structure loss. The cavities ranged from a simple occlusal cavity to mesio‐occlusal/disto‐occlusal (MO/DO) cavity with thick (≥2 mm) or thin (<2 mm) remaining axial walls, to a mesio‐occlusal‐distal (MOD) cavity regardless of the remaining axial wall thickness, and to structure loss beyond an MOD cavity. The different clinical situations obtained in the laboratory were organized in an ascending manner depending on the amount of tooth structure loss on a hypothetical scale (Figure 1). VT and ETT versions were included. A slightly similar hypothetical scale was suggested previously by Rocca and Krejci (2013).

A description of each cavity type in the hypothetical scale was extracted according to the amount of tooth structure loss and whether the tooth is vital or endodontically treated. Four types for VT and five types for ETT were prepared. The description of each type was as follows:

Type V1: A VT with an occlusal cavity and axial wall thickness of ≥2 mm.
Type E1: An ETT with an occlusal cavity and axial wall thickness of ≥2 mm.
Type V2: A VT with a MO/DO cavity and axial wall thickness of ≥2 mm.
Type E2: An ETT with a MO/DO cavity and axial wall thickness of ≥2 mm.
Type V3: A VT with a MO/DO cavity and axial wall thickness of <2 mm.
Type E3: An ETT with a MO/DO cavity and axial wall thickness of <2 mm.
Type V4: A VT with a MOD cavity regardless of the axial wall thickness.
Type E4: An ETT with a MOD cavity regardless of the axial wall thickness.
Type E5: An ETT with tooth structure loss beyond a MOD cavity.

**Figure 2:**
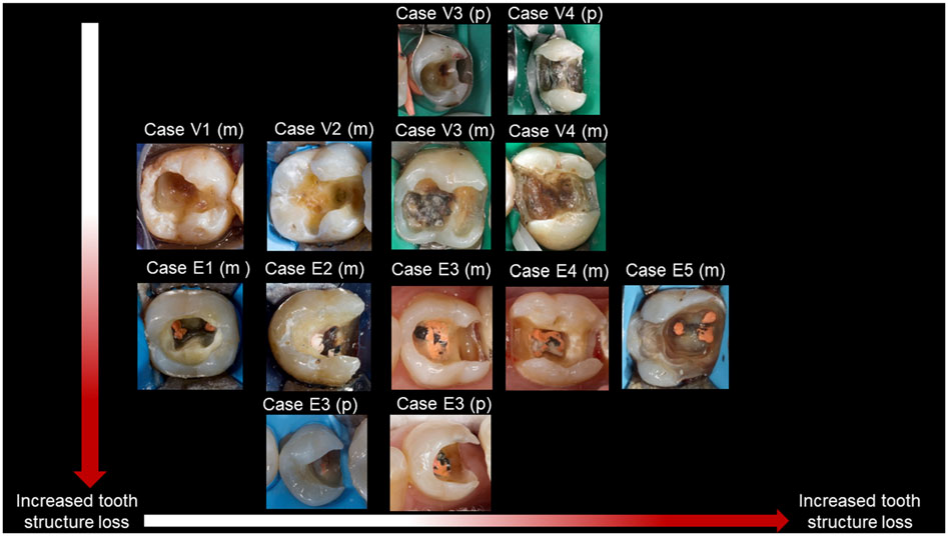
The clinical situations that were selected to simulate the hypothetical scale of different levels of tooth structure loss. The same numbering system used for the hypothetical scale in Figure 1 was used. V refers to a vital tooth, and E refers to an endodontically treated tooth. A letter (m)/(p) was added to refer to a molar/premolar variation

Thirteen clinical situations that simulated the hypothetical scale were then selected. Molar and premolar variations were selected for specific situations. The clinical situations selected were presented in Figure 2 in a manner similar to the hypothetical scale prepared in the laboratory.

The data collected were entered into an Excel spreadsheet and then transferred to the IBM SPSS Statistics software package (Corp IBM, 2013). The sample characteristics and different findings underwent descriptive analysis, and the results were presented in percentages.

Clinical situations in which 75% or more of the participants made the same decision were considered clinical situations with a high percentage of similarity in decision making. Clinical situations in which fewer than 75% of the participants made the same decision were considered clinical situations with a low percentage of similarity in decision making. The 75% cutoff point (three quarters of the participants) was selected arbitrarily. The “unsure” answer was considered a representation of the level of uncertainty of the best decision for that specific clinical situation. The associations between dentists' decisions and different variables were assessed using chi‐square test (*χ* ^2^).

## 3 RESULTS

One hundred twenty participants out of 182 agreed to participate in the study and submitted a complete response to the survey. This amounted to a response rate of 65.9%. The sample consisted of 120 dentists (70 females and 50 males). Their dental experience ranged from 1 to 28 years with a median of 3.5 years (interquartile range 1.1–10.8).

Descriptive analyses of the answers were carried out. The answers for each clinical situation were presented in Table 1. The analyses revealed a percentage of ≥75% in making the same decision for seven clinical situations. Of these clinical situations, five were for ETT, and only two were for VT. The highest percentage of similarity in decision making for ETT was for the molar tooth with structure loss beyond an MOD cavity (98% decided it needs cuspal coverage) and for VT was for the molar with an occlusal cavity and axial wall thickness of ≥2 mm (95% decided it does not need cuspal coverage). These two clinical situations corresponded to the two extremes of the hypothetical scale.

**Table 1:** Descriptive results of the participants' responses

Case description	Case reference number	Needs cuspal coverage (%)	Does not need cuspal coverage (%)	Unsure of the answer (%)	Similarity in decision making (%)
Vital teeth					
An occlusal cavity with axial wall thickness of ≥2 mm.	V1 (m)	5	95	—	≥75
A MO/DO cavity with axial wall thickness of ≥2 mm.	V2 (m)	16.7	76.7	6.7	≥75
A MO/DO cavity with axial wall thickness of <2 mm (molar).	V3 (m)	66.7	30	3.3	**<75**
A MO/DO cavity with axial wall thickness of <2 mm (premolar).	V3 (p)	33.3	55	11.7	**<75**
A MOD cavity regardless of the axial wall thickness (molar).	V4 (m)	56.7	16.7	26.7	**<75**
A MOD cavity regardless of the axial wall thickness (premolar).	V4 (p)	53.3	41.7	5	**<75**
Endodontically treated teeth					
An occlusal cavity with axial wall thickness of ≥2 mm.	E1 (m)	8.3	88.3	3.3	≥75
A MO/DO cavity with axial wall thickness of ≥2 mm (molar).	E2 (m)	71.7	26.7	1.7	**<75**
A MO/DO cavity with axial wall thickness of ≥2 mm (premolar).	E2 (p)	51.7	41.7	6.7	**<75**
A MO/DO cavity with axial wall thickness of <2 mm (molar).	E3 (m)	75	21.7	3.3	≥75
A MO/DO cavity with axial wall thickness of <2 mm (premolar).	E3 (p)	96.7	3.3	—	≥75
A MOD cavity regardless of the axial wall thickness.	E4 (m)	88.3	5	6.7	≥75
Tooth structure loss beyond an MOD cavity.	E5 (m)	98.3	1.7	—	≥75

Abbreviations: MOD, mesio‐occlusal‐distal; MO/DO, mesio‐occlusal/disto‐occlusal.

Bold emphasis, is to indicate the clinical situations in which less than 75% of the dentists agreed on a decision (i.e less than 75% of the dentists made similar decisions on whether cuspal coverage is needed or not).

For the majority of the clinical situations, the unsure answer was selected by less than 7% of the sample. However, two clinical situations of VT had a higher percentage of the unsure answer selected (the vital premolar with an MO/DO cavity and walls thickness of <2 mm, 11.7%, and the vital molar with an MOD cavity, 26.7%).

Three different categories of clinical situations were extracted from the analyses based on the 75% cutoff point and were presented in Table 2.

**Table 2:** Three different categories of clinical situations were extracted from the analyses based on the 75% cutoff point of the similarity in decision making

Categories based on the 75% cutoff point	Case reference	Percentage
Clinical situations in which ≥75% of dentists decided that cuspal coverage is needed		
	Case V1 (m)	95
	Case E1 (m)	88.3
	Case V2 (m)	76.6
Clinical situations in which <75% of dentists made similar decisions on whether cuspal coverage is needed or not		
	Case E2 (m)	71.7 “needs cuspal coverage”
	Case E2 (p)	51.7 “needs cuspal coverage”
	Case V3 (m)	66.7 “needs cuspal coverage”
	Case V3 (p)	55.0 “does not need cuspal coverage”
	Case V4 (m)	56.7 “needs cuspal coverage”
	Case V4 (p)	53.3 “needs cuspal coverage”
Clinical situations in which ≥75% of the dentists decided that cuspal coverage is needed		
	Case E3 (m)	75.0
	Case E3 (p)	96.7
	Case E4 (m)	88.3
	Case E5 (m)	98.3

A chi‐square test was performed in order to examine the association between choosing the “not sure” answer and the tooth condition (vital or endodontically treated), gender (female or male), and years of experience (≤5 or >5 years). The association was found to be statistically significant for the tooth condition (*P* < .001), whereas the association was found to be statistically insignificant for gender (*P* = .509) and years of experience (*P* = .223). Details of the analyses were presented in Table 3.

**Table 3:** Dentists' choice of the “not sure” answer according to the tooth condition (vital or endodontically treated), gender (female or male), and years of experience (≤5 or >5 years)

Dentists' decisions	VT No. (%)	ETT No. (%)	Females No. (%)	Males No. (%)	≤5 years No. (%)	>5 year No. (%)
A decision made	656 (91.1)	814 (96.9)	854 (93.8)	616 (94.8)	876 (93.6)	594 (95.2)
Not sure of decision	64 (8.9)	26 (3.1)	56 (6.2)	34 (5.2)	60 (6.4)	30 (4.8)
Total	720 (100)	840 (100)	910 (100)	650 (100)	936 (100)	624 (100)
*χ* ^2^ test	*χ* ^2^(1, *N*: 1560) = 22.884, *P* < .001*		*χ* ^2^(1, *N*: 1560) = 0.437, *P* = .509		*χ* ^2^(1, *N*: 1560) = 1.486, *P* = .223	

Abbreviations: ETT, endodontically treated teeth; VT, vital teeth.

* Statistically significant.

## 4 DISCUSSION

The current study targeted a random sample of dentists with different years of experience. Differences between dentists in decision making were observed. This is not unexpected, because many reports in the literature have reported significant differences between dentists' interpretations and treatment decision making in different areas of clinical dentistry (Bader & Shugars, 1993; Elderton, 1983; Elderton & Nuttall, 1983; Kay, Nuttall, & Kniil‐Jones, 1992; Mileman, Purdell‐Lewis, & Welle, 1982; Nuttall & Elderton, 1983). These differences could be a reflection of natural variability between dentists, or they could be a reflection of areas of uncertainty or disagreement in regard to the most suitable treatment option for a particular situation (Bader & Shugars, 1995; Shugars et al., 1997).

Evidence‐based decision making in treatment planning is essential for daily practice (Anderson, 2000). However, there is always the problem of accessibility and the difficulty of finding directly relevant clinical information (Al‐Ansari & ElTantawi, 2014; Rabe, Holmen, & Sjoegren, 2007). A recent online tool has been developed to aid dentists and students in making the best decision for restoring a single posterior tooth based on two main factors: whether the tooth is vital or not and based on the amount of tooth structure remaining. This tool could be useful for accessing a summary of the evidence for each situation, which would aid in decision making (Afrashtehfar & Tamimi, 2017). In the current study, the dentists were also asked to make their decision based on the vitality of the tooth and the amount of tooth structure remaining. The study identified clinical situations in which dissimilarity and uncertainty in decision making among dentists were observed.

The clinical situations selected for this study covered a wide range of scenarios that dentists encounter in their daily practice. The ascending hypothetical scale used to select those clinical situations represented a systematic increase in the amounts of tooth structure loss and could also be considered to represent an increase in the fracture risk of the tooth (Mondelli, Steagall, Ishikiriama, de Lima Navarro, & Soares, 1980; Rocca & Krejci, 2013).

It seemed that dentists were more inclined to provide an intracoronal restoration when the tooth had a minimal amount of structure loss and were more inclined to provide a cuspal coverage restoration when the tooth had a severe amount of structure loss (Shugars et al., 1997). It was the clinical situations lying in the middle of the scale with a moderate amount of structure loss that had more variability when it came to decision making.

The number of VT clinical situations that had lower similarity and higher uncertainty were more than the number of ETT clinical situations. Moreover, uncertainty in decision making were more likely to be reported for clinical situations of VT compared with clinical situations of ETT. This could be due to the lack of knowledge synthesis in the literature for deciding when cuspal coverage is needed for VT (Afrashtehfar, Emami, et al., 2017), compared with the available knowledge synthesis for ETT (Afrashtehfar, Ahmadi, et al., 2017; Goodacre & Spolnik, 1994; Smith & Schuman, 1997).

Dentists' experience and gender were not found to be significantly associated with uncertainty in decision making. The lack of significant difference could be due to the limited factors taken into account in the decision‐making process, which might have masked the differences within the sample. The decision‐making process could have also been affected by the dentists' work environment or place of training; however, this was not assessed in the current study (Burke & Lucarotti, 2009; Lucarotti et al., 2005a).

### 4.1 Clinical situations in which ≥75% of participants decided that cuspal coverage is not needed

The majority of the participants (95%) decided that there was no need for cuspal coverage for a VT with an occlusal cavity and remaining axial wall thickness of ≥2 mm. This decision was consistent with a recent systematic review (Afrashtehfar, Emami, et al., 2017), which found low failure rates in the studies that reported on the use of intracoronal restorations for such a clinical situation (Bernardo et al., 2007; Kopperud, Tveit, Gaarden, Sandvik, & Espelid, 2012; Lucarotti, Holder, & Burke, 2005b; Shi et al., 2010).

The majority of the participants (88.3%) decided that there was no need for cuspal coverage for ETT with an occlusal cavity and remaining axial wall thickness of ≥2 mm. In vitro studies support this decision (Reeh et al., 1989; Steele & Johnson, 1999). Reeh et al. (1989) detected a 20% reduction in tooth stiffness with an occlusal cavity and only a 5% reduction when a conservative endodontic access cavity was carried out. These results were confirmed by Steele and Johnson (1999). A recent systematic review found a 100% survival rate up to 6‐year follow‐up when full cuspal coverage restorations were used; however, their review did not include any studies that managed this clinical situation with intracoronal restorations (Afrashtehfar, Ahmadi, et al., 2017). One retrospective clinical study found endodontically treated molars with an occlusal cavity to have a survival rate of 78% at 5‐year follow‐up when restored with an intracoronal restoration (Nagasiri & Chitmongkolsuk, 2005). Randomized clinical trials comparing restorative treatment with and without cuspal coverage are needed for this clinical situation.

No need for cuspal coverage was reported by 76.6% of the participants for a VT with a MO/DO cavity and axial wall thickness of ≥2 mm. Studies reported good longevity for such clinical situation when managed with an intracoronal restoration without cuspal coverage (Afrashtehfar, Emami, et al., 2017; Opdam et al., 2014).

### 4.2 Clinical situations in which <75% of participants made similar decisions on whether cuspal coverage is needed or not

A low percentage of similarity in decision making appeared for an ETT with a MO/DO cavity and axial wall thickness of ≥2 mm (the need for cuspal coverage was decided by 71.7% for the molar and 51.7% for the premolar variation). The loss of one marginal ridge was found to lead to 45% reduction of tooth stiffness (Reeh et al., 1989). When a conservative endodontic access cavity was prepared, the stiffness was not significantly reduced (Reeh et al., 1989; Steele & Johnson, 1999). Other studies reported a twofold to a threefold increase of cuspal deflection when an endodontic access cavity was carried out for MO/DO and MOD cavities (Panitvisai & Messer, 1995). This was attributed to the increased depth associated with an access cavity preparation (Hansen & Asmussen, 1990) A retrospective study reported 80% survival rate over 3 years when intracoronal composite resin restoration was used for restoring ETT with a MO/DO cavity and axial wall thickness of >2.5 mm. The use of a fiber post with the intracoronal restoration was found to increase the survival rate to 95% (Scotti et al., 2015). One prospective clinical study reported no significant clinical difference between full cuspal coverage and intracoronal composite restorations for endodontically treated premolars with MO/DO cavities that had preserved cuspal structure over a 3‐year follow‐up (Mannocci et al., 2002). On the other hand, a systematic review suggested that ETT with MO/DO cavities should not be restored with intracoronal restorations because a lower failure rate (0% to 5.6%) was found when cuspal coverage was used (Ferrari et al., 2012; Mannocci et al., 2002; Signore, Kaitsas, Ravera, Angiero, & Benedicenti, 2011), compared with when intracoronal restorations were used (failure rate: 9% to 10.3%; Mannocci, Qualtrough, Worthington, Watson, & Pitt Ford, 2005). It is worth noting that the thickness of the remaining walls was not taken into consideration in some of the studies supporting this conclusion (Ferrari et al., 2012; Signore et al., 2011). Studies that included ETT with an MO/DO cavity and thick axial walls reported good survival without cuspal coverage (Mannocci et al., 2002; Scotti et al., 2015). However, more long‐term randomized clinical trials are needed to confirm the best treatment for this clinical situation.

A low percentage of similarity in decision making was found for the molar and premolar VT with a MO/DO cavity and thin axial walls (<2 mm). Studies reported that axial wall thickness of more than 2 mm would provide the tooth with good strength (Dietschi & Spreafico, 1997; Scotti et al., 2013). The wider the cavity preparation (thinner axial walls), the less the resistance to fracture in a tooth with a MO/DO cavity (Mondelli et al., 1980; Vale, 1959). From these studies, it could be suggested that a tooth with thin axial walls could be at higher risk of fracture than a tooth with thick axial walls. A partial cuspal coverage of the thin axial wall could be an option. A systematic review found no clinical studies of managing this clinical situation with a crown and suggested that providing cuspal coverage using a crown could be considered an overtreatment and would be hard to justify (Afrashtehfar, Emami, et al., 2017).

A low percentage of similarity in decision making was also reported for the molar and premolar VT with a MOD cavity. Fewer than 60% decided that this situation requires cuspal coverage, and 26.7% of the participants were unsure whether the molar variation required cuspal coverage or not. The marginal ridges were proven to be critical in the maintenance of the tooth stiffness and limiting excessive cuspal deflection and movement (González‐López, De Haro‐Gasquet, Vilchez‐Diaz, Ceballos, & Bravo, 2006; Lin, Chang, & Liu, 2008; Linn & Messer, 1994; Pantvisai & Messer, 1995; Reeh et al., 1989; Salameh et al., 2006). A loss of 63% of tooth stiffness was reported for a MOD cavity with or without an access cavity preparation (Reeh et al., 1989). Steele and Johnson (1999) noted that a tooth with a MOD cavity had lower resistance to fracture than a tooth with an endodontic access cavity alone. It could be suggested that a tooth that lost both of its marginal ridges is at an increased risk of fracture and would benefit from cuspal coverage even if not endodontically treated. Conservative MOD cavities in depth and width in VT could be considered an exemption (Reagan, Schwandt, & Duncanson, 1989). On the other hand, a recent systematic review reported that no information from clinical studies were found in the literature regarding providing cuspal coverage for VT with MOD cavities, and therefore, the treatment of choice suggested was an intracoronal restoration (Afrashtehfar, Emami, et al., 2017). It is worth mentioning that more conservative methods for cuspal coverage are currently available (Rocca & Krejci, 2013), such as adhesive direct or indirect onlays (Murphy, McDonald, Petrie, Palmer, & Setchell, 2009). These adhesive conservative methods in providing cuspal coverage could justify providing cuspal coverage in such clinical situations when fear of fracture of the tooth exists.

### 4.3 Clinical situations in which ≥75% of the participants decided that cuspal coverage is needed

A high percentage of the participants decided the need for cuspal coverage for ETT with a MO/DO cavity and thin axial walls (75% for molar and 96.7% for the premolar). This decision was consistent with a systematic review in which the failure rate was found to be reduced when such clinical situations were managed using cuspal coverage instead of using intracoronal restorations (Afrashtehfar, Ahmadi, et al., 2017). Scotti et al. (2013) found the remaining wall thickness to be a very important clinical parameter in the resistance of fracture in endodontically treated premolars and suggested that an axial wall thickness of less than 2 mm is an indication for cuspal coverage to improve the fracture resistance in maxillary premolars. Other in vitro studies have reported a reduction in tooth resistance to fracture with a wider isthmus preparation in MO/DO and MOD cavities (Mondelli et al., 1980; Reagan et al., 1989) and with a marginal ridge thicknesses of less than 1 mm in ETT (Shahrbaf, Mirzakouchaki, Oskoui, & Kahnamoui, 2007).

A high percentage of the participants decided the need for cuspal coverage for an ETT with an MOD cavity (88.3%) and for an ETT with structure loss beyond an MOD cavity (98.3%). As discussed previously, significant loss of fracture resistance and increased cuspal deflection are associated with ETT with MOD cavities (Hansen & Asmussen, 1990; Pantvisai & Messer, 1995; Reeh et al., 1989; Steele & Johnson, 1999). These teeth would certainly benefit from cuspal coverage. Cuspal coverage for ETT with structure loss beyond a MOD cavity would not only be for the protection of the remaining tooth structure but also for facilitating the reestablishment of the lost occlusal anatomy. No clinical situations of VT with structure loss beyond a MOD cavity were included in the current study. However, studies have recommended full cuspal coverage for VT with one remaining axial wall (Afrashtehfar, Emami, et al., 2017).

Management of an ETT with cuspal coverage using a crown would generally provide better protection for the tooth and, as mentioned previously, is supported by many studies from the literature. However, it is not without a biological cost (Edelhoff & Sorensen, 2002). Avoiding a full cuspal coverage crown would have many advantages when provided for teeth with a sufficient amount of tooth structure remaining, such as preserving tooth structure and reducing the time and cost of the treatment (Fedorowicz et al., 2012; Shugars et al., 1997).

From the discussion of the clinical situations selected according to the hypothetical scale, three different categories could be suggested to help the dentist in decision making regarding the need for cuspal coverage:

- Minimally destructed teeth include VT and ETT teeth with an occlusal cavity or a MO/DO cavity and thick axial walls (≥2 mm). This category does not generally need cuspal coverage. However, studies are needed to confirm this decision for ETT.
- Moderately destructed teeth include VT and ETT teeth with a MO/DO cavity with thin axial walls (<2 mm) or a MOD cavity. In this category, cuspal coverage is needed for ETT, whereas it might be avoided for the VT.
- Severely destructed teeth include ETT and VT with structure loss beyond an MOD cavity. This category needs cuspal coverage.

An important limitation of this study was that it only assessed the need for cuspal coverage, according to dentists' opinions, depending on the amount of tooth structure remaining without taking into account the type of the patients' occlusion, parafunctional habits, or the amount and direction of occlusal forces on the teeth.

Parafunctional habits such as bruxism can generate a significant amount of forces on teeth (Nishigawa et al., 2001). This factor should be taken into consideration when a decision is made about the most appropriate treatment option available in regard to the need for cuspal coverage and the material to be selected.

A tooth that is under axial and lateral forces will have to withstand more forces than a tooth that is only subjected to axial forces (Loney et al., 1995; Torbjörner & Fransson, 2004). When testing premolars using different loading directions, premolars that were subjected to oblique occlusal loads were at more risk of fracture than those subjected to axial occlusal loads (Palamara, Palamara, Tyas, & Messer, 2000; Zhu, Rong, Wang, & Gao, 2017). This factor should also be considered when assessing the restorative options for a posterior tooth, because it could be a crucial factor for the longevity of the restoration and the endodontically treated tooth (Krejci, Duc, Dietschi, & de Campos, 2003; Uyehara, Davis, & Overton, 1999).

The patient factors should also be considered (Demarco, Corrêa, Cenci, Moraes, & Opdam, 2012), and decision making should take into account the patient's caries levels, health, and financial ability.

The clinical cases used in the current study were a combination of molar and premolar teeth. Whether decision making for molar teeth compared with premolar teeth with the same amount of tooth structure loss would differ should be addressed in future studies.

## 5 CONCLUSIONS

Assessment of dentists' decision making regarding the need for cuspal coverage based on tooth vitality and the amount of tooth structure loss was carried out. Dissimilarities in decision making and uncertainty regarding the need for cuspal coverage were observed among dentists, especially for VT and teeth with a moderate amount of structure loss.

## CONFLICT OF INTEREST

This research does not involve any conflict of interests.

## ETHICAL APPROVAL

The study was granted an ethical approval with a favorable opinion by The Deanship of Academic Research at the University of Jordan in Amman, Jordan.

## References

[cre2185-bib-0001] Afrashtehfar, K. I. , Ahmadi, M. , Emami, E. , Abi‐Nader, S. , & Tamimi, F. (2017). Failure of single‐unit restorations on root filled posterior teeth: A systematic review. International Endodontic Journal, 50, 951–966. 27870102 10.1111/iej.12723

[cre2185-bib-0002] Afrashtehfar, K. I. , Emami, E. , Ahmadi, M. , Eilayyan, O. , Abi‐Nader, S. , & Tamimi, F. (2017). Failure rate of single‐unit restorations on posterior vital teeth: A systematic review. The Journal of Prosthetic Dentistry, 117, 345–353. 27765400 10.1016/j.prosdent.2016.08.003

[cre2185-bib-0003] Afrashtehfar, K. I. , & Tamimi, F. (2017). An online tool that provides access to evidence‐based literature on dental restorations: www.crownorfill.com. The Journal of Prosthetic Dentistry, 118, 696–697. 28461049 10.1016/j.prosdent.2017.02.001

[cre2185-bib-0004] Al‐Ansari, A. , & ElTantawi, M. (2014). Factors affecting self‐reported implementation of evidence‐based practice among a group of dentists. The Journal of Evidence‐Based Dental Practice, 14, 2–8. 10.1016/j.jebdp.2013.11.001 24581703

[cre2185-bib-0005] Anderson, J. D. (2000). Need for evidence‐based practice in prosthodontics. The Journal of Prosthetic Dentistry, 83, 58–65. 10633023 10.1016/s0022-3913(00)70089-8

[cre2185-bib-0006] Aquilino, S. A. , & Caplan, D. J. (2002). Relationship between crown placement and the survival of endodontically treated teeth. The Journal of Prosthetic Dentistry, 87, 256–263. 11941351 10.1067/mpr.2002.122014

[cre2185-bib-0007] Assif, D. , & Gorfil, C. (1994). Biomechanical considerations in restoring endodontically treated teeth. The Journal of Prosthetic Dentistry, 71, 565–567. 8040817 10.1016/0022-3913(94)90438-3

[cre2185-bib-0008] Bader, J. D. , & Shugars, D. A. (1993). Agreement among dentists' recommendations for restorative treatment. Journal of Dental Research, 72, 891–896. 10.1177/00220345930720051001 8501287

[cre2185-bib-0009] Bader, J. D. , & Shugars, D. A. (1995). Variation in dentists' clinical decisions. Journal of Public Health Dentistry, 55, 181–188. 10.1111/j.1752-7325.1995.tb02364.x 7562733

[cre2185-bib-0010] Bernardo, M. , Luis, H. , Martin, M. D. , Leroux, B. G. , Rue, T. , Leitão, J. , & DeRouen, T. A. (2007). Survival and reasons for failure of amalgam versus composite posterior restorations placed in a randomized clinical trial. Journal of the American Dental Association (1939), 138, 775–783. 10.14219/jada.archive.2007.0265 17545266

[cre2185-bib-0011] Burke, F. J. T. , & Lucarotti, P. S. K. (2009). Ten‐year outcome of crowns placed within the General Dental Services in England and Wales. Journal of Dentistry, 37, 12–24. 10.1016/j.jdent.2008.03.017 18487003

[cre2185-bib-0012] Cheung, W. (2005). A review of the management of endodontically treated teeth: Post, core and the final restoration. Journal of the American Dental Association (1939), 136, 611–619. 10.14219/jada.archive.2005.0232 15966648

[cre2185-bib-0013] Corp IBM (2013). IBM SPSS statistics for windows, version 22.0. Armonk, NY: IBM Corp.

[cre2185-bib-0014] Demarco, F. F. , Corrêa, M. B. , Cenci, M. S. , Moraes, R. R. , & Opdam, N. J. M. (2012). Longevity of posterior composite restorations: Not only a matter of materials. Dental Materials, 28, 87–101. 10.1016/j.dental.2011.09.003 22192253

[cre2185-bib-0015] Dietschi, D. , & Spreafico, R. (1997). Adhesive metal‐free restorations: Current concepts for the esthetic treatment of posterior teeth. IL: Quintessence Publishing.

[cre2185-bib-0016] Edelhoff, D. , & Sorensen, J. A. (2002). Tooth structure removal associated with various preparation designs for anterior teeth. The Journal of Prosthetic Dentistry, 87, 503–509. 10.1067/mpr.2002.124094 12070513

[cre2185-bib-0017] Elderton, R. J. (1983). Longitudinal study of dental treatment in the general dental service in Scotland. British Dental Journal, 155, 91–96. 10.1038/sj.bdj.4805135 6577903

[cre2185-bib-0018] Elderton, R. J. , & Nuttall, N. M. (1983). Variation among dentists in planning treatment. British Dental Journal, 154, 201–206. 10.1038/sj.bdj.4805041 6573898

[cre2185-bib-0019] Fedorowicz, Z. , Carter, B. , de Souza, R. F. , de Andrade Lima Chaves, C. , Nasser, M. , & Sequeira‐Byron, P. (2012). Single crowns versus conventional fillings for the restoration of root filled teeth. Cochrane Database of Systematic Reviews, 5, CD009109 10.1002/14651858.CD009109.pub2 22592736

[cre2185-bib-0020] Ferrari, M. , Vichi, A. , Fadda, G. M. , Cagidiaco, M. C. , Tay, F. R. , Breschi, L. , … Goracci, C. (2012). A randomized controlled trial of endodontically treated and restored premolars. Journal of Dental Research, 91, 72–78. 10.1177/0022034512447949 22699672

[cre2185-bib-0021] González‐López, S. , De Haro‐Gasquet, F. , Vilchez‐Diaz, M. A. , Ceballos, L. , & Bravo, M. (2006). Effect of restorative procedures and occlusal loading on cuspal deflection. Operative Dentistry, 31, 33–38. 10.2341/04-165 16536191

[cre2185-bib-0022] Goodacre, C. J. , & Spolnik, K. J. (1994). The prosthodontic management of endodontically treated teeth: A literature review. Part I. Success and failure data, treatment concepts. Journal of Prosthodontics, 3, 243–250. 10.1111/j.1532-849X.1994.tb00162.x 7866508

[cre2185-bib-0023] Grembowski, D. , Fiset, L. , Milgrom, P. , Forrester, K. , & Spadafora, A. (1997). Factors influencing the appropriateness of restorative dental treatment: An epidemiologic perspective. Journal of Public Health Dentistry, 57, 19–30. 10.1111/j.1752-7325.1997.tb02469.x 9150060

[cre2185-bib-0024] Hansen, E. K. , & Asmussen, E. (1990). In vivo fractures of endodontically treated posterior teeth restored with enamel‐bonded resin. Dental Traumatology, 6, 218–225. 10.1111/j.1600-9657.1990.tb00422.x 2133313

[cre2185-bib-0025] Hansen, E. K. , Asmussen, E. , & Christiansen, N. C. (1990). In vivo fractures of endodontically treated posterior teeth restored with amalgam. Dental Traumatology, 6, 49–55. 10.1111/j.1600-9657.1990.tb00389.x 2132209

[cre2185-bib-0026] Huang, T.‐J. G. , Schilder, H. , & Nathanson, D. (1992). Effects of moisture content and endodontic treatment on some mechanical properties of human dentin. Journal of Endodontia, 18, 209–215. 10.1016/S0099-2399(06)81262-8 1402574

[cre2185-bib-0027] Kay, E. J. , Nuttall, N. M. , & Kniil‐Jones, R. (1992). Restorative treatment thresholds and agreement in treatment decision‐making. Community Dentistry and Oral Epidemiology, 20, 265–268. 10.1111/j.1600-0528.1992.tb01696.x 1424545

[cre2185-bib-0028] Kopperud, S. E. , Tveit, A. B. , Gaarden, T. , Sandvik, L. , & Espelid, I. (2012). Longevity of posterior dental restorations and reasons for failure. European Journal of Oral Sciences, 120, 539–548. 10.1111/eos.12004 23167471

[cre2185-bib-0029] Krejci, I. , Duc, O. , Dietschi, D. , & de Campos, E. (2003). Marginal adaptation, retention and fracture resistance of adhesive composite restorations on devital teeth with and without posts. Operative Dentistry, 28, 127–135. 12670067

[cre2185-bib-0030] Lin, C.‐L. , Chang, Y.‐H. , & Liu, P.‐R. (2008). Multi‐factorial analysis of a cusp‐replacing adhesive premolar restoration: A finite element study. Journal of Dentistry, 36, 194–203. 10.1016/j.jdent.2007.11.016 18221831

[cre2185-bib-0031] Linn, J. , & Messer, H. H. (1994). Effect of restorative procedures on the strength of endodontically treated molars. Journal of Endodontia, 20, 479–485. 10.1016/S0099-2399(06)80043-9 7714419

[cre2185-bib-0032] Loney, R. W. , Moulding, M. B. , & Ritsco, R. G. (1995). The effect of load angulation on fracture resistance of teeth restored with cast post and cores and crowns. The International Journal of Prosthodontics, 8, 247–251. 10348593

[cre2185-bib-0033] Lucarotti, P. S. K. , Holder, R. L. , & Burke, F. J. T. (2005a). Outcome of direct restorations placed within the general dental services in England and Wales (Part 3): Variation by dentist factors. Journal of Dentistry, 33, 827–835. 10.1016/j.jdent.2005.03.009 16246480

[cre2185-bib-0034] Lucarotti, P. S. K. , Holder, R. L. , & Burke, F. J. T. (2005b). Analysis of an administrative database of half a million restorations over 11 years. Journal of Dentistry, 33, 791–803. 10.1016/j.jdent.2005.06.011 16214285

[cre2185-bib-0035] Mannocci, F. , Bertelli, E. , Sherriff, M. , Watson, T. F. , & Ford, T. R. P. (2002). Three‐year clinical comparison of survival of endodontically treated teeth restored with either full cast coverage or with direct composite restoration. The Journal of Prosthetic Dentistry, 88, 297–301. 10.1067/mpr.2002.128492 12426500

[cre2185-bib-0036] Mannocci, F. , Qualtrough, A. J. , Worthington, H. V. , Watson, T. F. , & Pitt Ford, T. R. (2005). Randomized clinical comparison of endodontically treated teeth restored with amalgam or with fiber posts and resin composite: Five‐year results. Operative Dentistry, 30, 9–15. 15765952

[cre2185-bib-0037] Mileman, P. , Purdell‐Lewis, D. , & Welle, L. (1982). Variation in radiographic caries diagnosis and treatment decisions among university teachers. Community Dentistry and Oral Epidemiology, 10, 329–334. 10.1111/j.1600-0528.1982.tb00404.x 6961983

[cre2185-bib-0038] Mondelli, J. , Steagall, L. , Ishikiriama, A. , de Lima Navarro, M. F. , & Soares, F. B. (1980). Fracture strength of human teeth with cavity preparations. The Journal of Prosthetic Dentistry, 43, 419–422. 10.1016/0022-3913(80)90213-9 6928479

[cre2185-bib-0039] Morgano, S. M. , Hashem, A. F. , Fotoohi, K. , & Rose, L. (1994). A nationwide survey of contemporary philosophies and techniques of restoring endodontically treated teeth. The Journal of Prosthetic Dentistry, 72, 259–267. 10.1016/0022-3913(94)90339-5 7965899

[cre2185-bib-0040] Murphy, F. , McDonald, A. , Petrie, A. , Palmer, G. , & Setchell, D. (2009). Coronal tooth structure in root‐treated teeth prepared for complete and partial coverage restorations. Journal of Oral Rehabilitation, 36, 451–461. 10.1111/j.1365-2842.2009.01952.x 19422436

[cre2185-bib-0041] Nagasiri, R. , & Chitmongkolsuk, S. (2005). Long‐term survival of endodontically treated molars without crown coverage: A retrospective cohort study. The Journal of Prosthetic Dentistry, 93, 164–170. 10.1016/j.prosdent.2004.11.001 15674228

[cre2185-bib-0042] Nishigawa, K. , Bando, E. , & Nakano, M. (2001). Quantitative study of bite force during sleep associated bruxism. Journal of Oral Rehabilitation, 28, 485–491. 10.1046/j.1365-2842.2001.00692.x 11380790

[cre2185-bib-0043] Nuttall, N. M. , & Elderton, R. J. (1983). The nature of restorative dental treatment decisions. British Dental Journal, 154, 363–365. 10.1038/sj.bdj.4805093 6575799

[cre2185-bib-0044] Opdam, N. J. M. , Van De Sande, F. H. , Bronkhorst, E. , Cenci, M. S. , Bottenberg, P. , Pallesen, U. , … van Dijken, J. W. (2014). Longevity of posterior composite restorations: A systematic review and meta‐analysis. Journal of Dental Research, 93, 943–949. 10.1177/0022034514544217 25048250 PMC4293707

[cre2185-bib-0045] Palamara, D. , Palamara, J. E. A. , Tyas, M. J. , & Messer, H. H. (2000). Strain patterns in cervical enamel of teeth subjected to occlusal loading. Dental Materials, 16, 412–419. 10.1016/S0109-5641(00)00036-1 10967190

[cre2185-bib-0046] Panitvisai, P. , & Messer, H. H. (1995). Cuspal deflection in molars in relation to endodontic and restorative procedures. Journal of Endodontia, 21, 57–61. 10.1016/S0099-2399(06)81095-2 7714437

[cre2185-bib-0047] Pantvisai, P. , & Messer, H. H. (1995). Cuspal deflection in molars in relation to endodontic and restorative procedures. Journal of Endodontia, 21, 57–61. 10.1016/S0099-2399(06)81095-2 7714437

[cre2185-bib-0048] Rabe, P. , Holmen, A. , & Sjoegren, P. (2007). Attitudes, awareness and perceptions on evidence based dentistry and scientific publications among dental professionals in the county of Halland, Sweden: A questionnaire survey. Swedish Dental Journal, 31, 113–120. 17970167

[cre2185-bib-0049] Randow, K. , & Glantz, P.‐O. (1986). On cantilever loading of vital and non‐vital teeth an experimental clinical study. Acta Odontologica Scandinavica, 44, 271–277. 10.3109/00016358609004733 3544657

[cre2185-bib-0050] Rasines Alcaraz, M. G. , Veitz‐Keenan, A. , Sahrmann, P. , Schmidlin, P. R. , Davis, D. , & Iheozor‐Ejiofor, Z. (2014). Direct composite resin fillings versus amalgam fillings for permanent or adult posterior teeth. Cochrane Database of Systematic Reviews, (3). 10.1002/14651858.CD005620.pub2 24683067

[cre2185-bib-0051] Reagan, S. E. , Schwandt, N. W. , & Duncanson, M. G. Jr. (1989). Fracture resistance of wide‐isthmus mesio‐occlusodistal preparations with and without amalgam cuspal coverage. Quintessence International, 20, 469–472. 2626535

[cre2185-bib-0052] Reeh, E. S. , Messer, H. H. , & Douglas, W. H. (1989). Reduction in tooth stiffness as a result of endodontic and restorative procedures. Journal of Endodontia, 15, 512–516. 10.1016/S0099-2399(89)80191-8 2639947

[cre2185-bib-0053] Robbins, J. W. (1990). Guidelines for the restoration of endodontically treated teeth. Journal of the American Dental Association (1939), 120, 558–566. 10.14219/jada.archive.1990.0087 2186075

[cre2185-bib-0054] Rocca, G. T. , & Krejci, I. (2013). Crown and post‐free adhesive restorations for endodontically treated posterior teeth: From direct composite to endocrowns. The European Journal of Esthetic Dentistry, 8, 156–179. 23712338

[cre2185-bib-0055] Salameh, Z. , Sorrentino, R. , Papacchini, F. , Ounsi, H. F. , Tashkandi, E. , Goracci, C. , & Ferrari, M. (2006). Fracture resistance and failure patterns of endodontically treated mandibular molars restored using resin composite with or without translucent glass fiber posts. Journal of Endodontia, 32, 752–755. 10.1016/j.joen.2006.02.002 16861075

[cre2185-bib-0056] Scotti, N. , Eruli, C. , Comba, A. , Paolino, D. S. , Alovisi, M. , Pasqualini, D. , & Berutti, E. (2015). Longevity of class 2 direct restorations in root‐filled teeth: A retrospective clinical study. Journal of Dentistry, 43, 499–505. 10.1016/j.jdent.2015.02.006 25701467

[cre2185-bib-0057] Scotti, N. , Rota, R. , Scansetti, M. , Paolino, D. S. , Chiandussi, G. , Pasqualini, D. , & Berutti, E. (2013). Influence of adhesive techniques on fracture resistance of endodontically treated premolars with various residual wall thicknesses. The Journal of Prosthetic Dentistry, 110, 376–382. 10.1016/j.prosdent.2013.08.001 24095213

[cre2185-bib-0058] Sedgley, C. M. , & Messer, H. H. (1992). Are endodontically treated teeth more brittle? Journal of Endodontia, 18, 332–335. 10.1016/S0099-2399(06)80483-8 1402595

[cre2185-bib-0059] Shahrbaf, S. , Mirzakouchaki, B. , Oskoui, S. S. , & Kahnamoui, M. A. (2007). The effect of marginal ridge thickness on the fracture resistance of endodontically‐treated, composite restored maxillary premolars. Operative Dentistry, 32, 285–290. 10.2341/06-83 17555181

[cre2185-bib-0060] Shi, L. , Wang, X. , Zhao, Q. , Zhang, Y. , Zhang, L. , Ren, Y. , & Chen, Z. (2010). Evaluation of packable and conventional hybrid resin composites in Class I restorations: Three‐year results of a randomized, double‐blind and controlled clinical trial. Operative Dentistry, 35, 11–19. 10.2341/09-027CR 20166406

[cre2185-bib-0061] Shugars, D. , Hayden, W. , Crall, J. , & Scurria, M. (1997). Variation in the use of crowns and their alternatives. Journal of Dental Education, 61, 22–28. 9024339

[cre2185-bib-0062] Signore, A. , Kaitsas, V. , Ravera, G. , Angiero, F. , & Benedicenti, S. (2011). Clinical evaluation of an oval‐shaped prefabricated glass fiber post in endodontically treated premolars presenting an oval root canal cross‐section: A retrospective cohort study. The International Journal of Prosthodontics, 24, 255–263. 21519574

[cre2185-bib-0063] Smith, C. T. , & Schuman, N. (1997). Restoration of endodontically treated teeth: A guide for the restorative dentist. Quintessence International, 28, 457–462. 9477895

[cre2185-bib-0064] Sorensen, J. A. , & Martinoff, J. T. (1984). Intracoronal reinforcement and coronal coverage: A study of endodontically treated teeth. The Journal of Prosthetic Dentistry, 51, 780–784. 10.1016/0022-3913(84)90376-7 6376780

[cre2185-bib-0065] Steele, A. , & Johnson, B. R. (1999). In vitro fracture strength of endodontically treated premolars. Journal of Endodontia, 25, 6–8. 10.1016/S0099-2399(99)80389-6 10196835

[cre2185-bib-0066] Torbjörner, A. , & Fransson, B. (2004). Biomechanical aspects of prosthetic treatment of structurally compromised teeth. The International Journal of Prosthodontics, 17, 135–141. 15119862

[cre2185-bib-0067] Uyehara, M. Y. , Davis, R. D. , & Overton, J. D. (1999). Cuspal reinforcement in endodontically treated molars. Operative Dentistry, 24, 364–370. 10823086

[cre2185-bib-0068] Vale, W. A. (1959). Cavity preparation and further thoughts on high speed. British Dental Journal, 107, 333–340.

[cre2185-bib-0069] Zarow, M. , Devoto, W. , & Saracinelli, M. (2009). Reconstruction of endodontically treated posterior teeth—With or without post? Guidelines for the dental practitioner. The European Journal of Esthetic Dentistry, 4. 20111757

[cre2185-bib-0070] Zhu, J. , Rong, Q. , Wang, X. , & Gao, X. (2017). Influence of remaining tooth structure and restorative material type on stress distribution in endodontically treated maxillary premolars: A finite element analysis. The Journal of Prosthetic Dentistry, 117, 646–655. 10.1016/j.prosdent.2016.08.023 27881319

